# Prevalence, awareness, treatment and control of hypertension and sodium intake in Zhejiang Province, China: A cross-sectional survey in 2017

**DOI:** 10.1371/journal.pone.0226756

**Published:** 2019-12-23

**Authors:** Xiaofu Du, Le Fang, Jianwei Xu, Xiangyu Chen, Jie Zhang, Yamin Bai, Jing Wu, Jixiang Ma, Min Yu, Jieming Zhong

**Affiliations:** 1 Department of Non-Communicable Disease Control and Prevention, Zhejiang Provincial Center for Disease Control and Prevention, Hangzhou, Zhejiang, P. R. China; 2 National Center for Chronic and Noncommunicable Disease Control and Prevention, Chinese Center for Disease Control and Prevention, Beijing, P. R. China; 3 Division of Non-communicable Disease Control and Community Health, Chinese Center for Disease Control and Prevention, Beijing, P. R. China; International University of Health and Welfare, School of Medicine, JAPAN

## Abstract

**Background:**

In recent decades, hypertension has become a popular public health problem in China. In 2017, a cross-sectional survey about hypertension and sodium intake measured by 24-hour (24-h) urine was launched in Zhejiang Province, to provide the basis of the Chinese population to develop a salt reduction intervention and prevention of hypertension strategy.

**Methods:**

Cross-sectional data were obtained from 7512 participants aged 18 to 69 years in Zhejiang Province of China by complex, multistage sampling methods. The survey included face-to-face questionnaires and physical examination among all participants. Blood pressures and the use of anti-hypertension medications were used to determine hypertension. One thousand five hundred participants of them were asked to collect 24-h urine to measure sodium and potassium levels to assess intake. All rates and means were weighted by sampling weight and population structure of the province.

**Results:**

The weighted means of systolic blood pressure (SBP) and diastolic blood pressure (DBP) were 127.78 mm Hg (95% confidence interval [CI], 127.21–128.36) and 79.40 mm Hg (95%CI, 79.04–79.76). The weighted hypertension prevalence was 30.41% (95%CI, 28.91–31.91). Among those classified as having hypertension, 43.55% (95%CI, 40.77–46.34) were aware of the fact that they were suffering from hypertension, 32.05% (95%CI, 29.49–34.61) of them reported taking anti-hypertension medications, only 14.48% (95%CI, 12.54–16.42) had their blood pressure controlled. The weighted means of 24-h urinary sodium was 165.52 mmol (standard deviation [SD], 2.92), representing that the mean intake of sodium chloride was 9.68g (SD, 0.17) through conversion.

**Conclusion:**

These cross-sectional survey results show that hypertension and excessive sodium intake in adults are prevalent in Zhejiang Province, China. Salt reduction and prevention of hypertension is still an urgent public health work.

## Introduction

Hypertension is a severe public health problem for both China and all over the world, which not only has a high prevalence but also increases the incidence of cardiovascular disease and kidney disease [1]. The prevalence of hypertension increased from 18.8% to 23.2% by the Chinese national nutrition and health survey in 2002 and the China Hypertension Survey 2012–2015, which means that there is one hypertensive patient per four people [2, 3]. The results of the two surveys found that the awareness rate and antihypertensive medication rate of hypertension increased significantly during the decade, while the control rate decreased. Hypertension is a key but modifiable risk factor for cardiovascular disease, while hypertension prevention and control tasks are still very challenging in China [4].

Several known factors increase blood pressure, such as high-salt diets, obesity, alcohol consumption, smoking, lack of exercise, and mental stress [5–7]. Among them, high-level salt diets are one of the most important factors [8] and also associated with cardiovascular disease. Studies have shown that excessive sodium intake is associated with elevated blood pressure, and an appropriate increase in potassium intake helps lower blood pressure, and the ratio of sodium to potassium is also associated with blood pressure[9–11]. Since sodium intake has a significant effect on blood pressure, the population’s sodium intake is increasingly being monitored [6, 12]. Researches on sodium intake mainly focus on dietary recall or records from 24 to 96 hours, food frequency questionnaires, or 24-h urinary sodium detection. Generally, 24-h urinary sodium is the gold standard for estimating population salt intake. We collected the 24-h urine of some respondents and tested the indicators such as sodium and potassium to obtain the salt intake level of the population accurately.

Located in eastern China, Zhejiang Province is a relatively economically developed province with a current resident population of 55 million. In 2017, we launched a cross-sectional survey relying on provincial and county-level Center for Disease Control and Prevention (CDC) and grassroots township hospitals/community health service centers, which aimed to develop and identify priority interventions like sodium reduction to prevent hypertension in the Zhejiang. This survey is also the first large-scale collection of 24-h urine in Zhejiang Province, which is relatively rare in China. It can rule out the survey bias of dietary review to obtain reliable data.

## Method

### Sample size and sampling frame

Conducted between December 2016 and May 2017, the survey was a population-based cross-sectional survey of residents aged 18 to 69 years. Based on the calculation formula of sample size:

$$n=deff\left(\frac{\mu_{a}^{2}\times \pi (1‐\pi )}{\delta^{2}}\right)$$

The required sample size to estimate the population prevalence of hypertension was 7500. For salt intake estimation:

$$n={\left(\frac{\mu_{\alpha }\sigma }{\delta }\right)}^{2}$$

The required sample size was 1500. Participants without disability and mental disorders living in the selected areas for 6 to 12 months before the investigation, were eligible to participate in the survey. We used complex, 4-stage cluster sampling to select the participants. Firstly, we selected five counties/districts from 89 counties and districts after stratification by geographic distribution and by residence status. Secondly, using proportional probability sampling, we selected three townships (in rural areas) or two streets (in urban areas) from each selected county or district. Then, also using the same way, we selected five villages (in rural areas) or neighborhood communities (in urban areas) from each sampled township (rural) or street (urban). Finally, we randomly selected one hundred adults from the residents’ list in each selected village and neighborhood community. We collected a total of 7512 participants aged 18 to 69 years among 75 villages and communities. In each village or neighborhood committee, two subjects were randomly selected from each layer (two-level gender and five-layer age), and finally, 1499 subjects were collected for 24-h urine.

### Main measures

**Fig 1:**
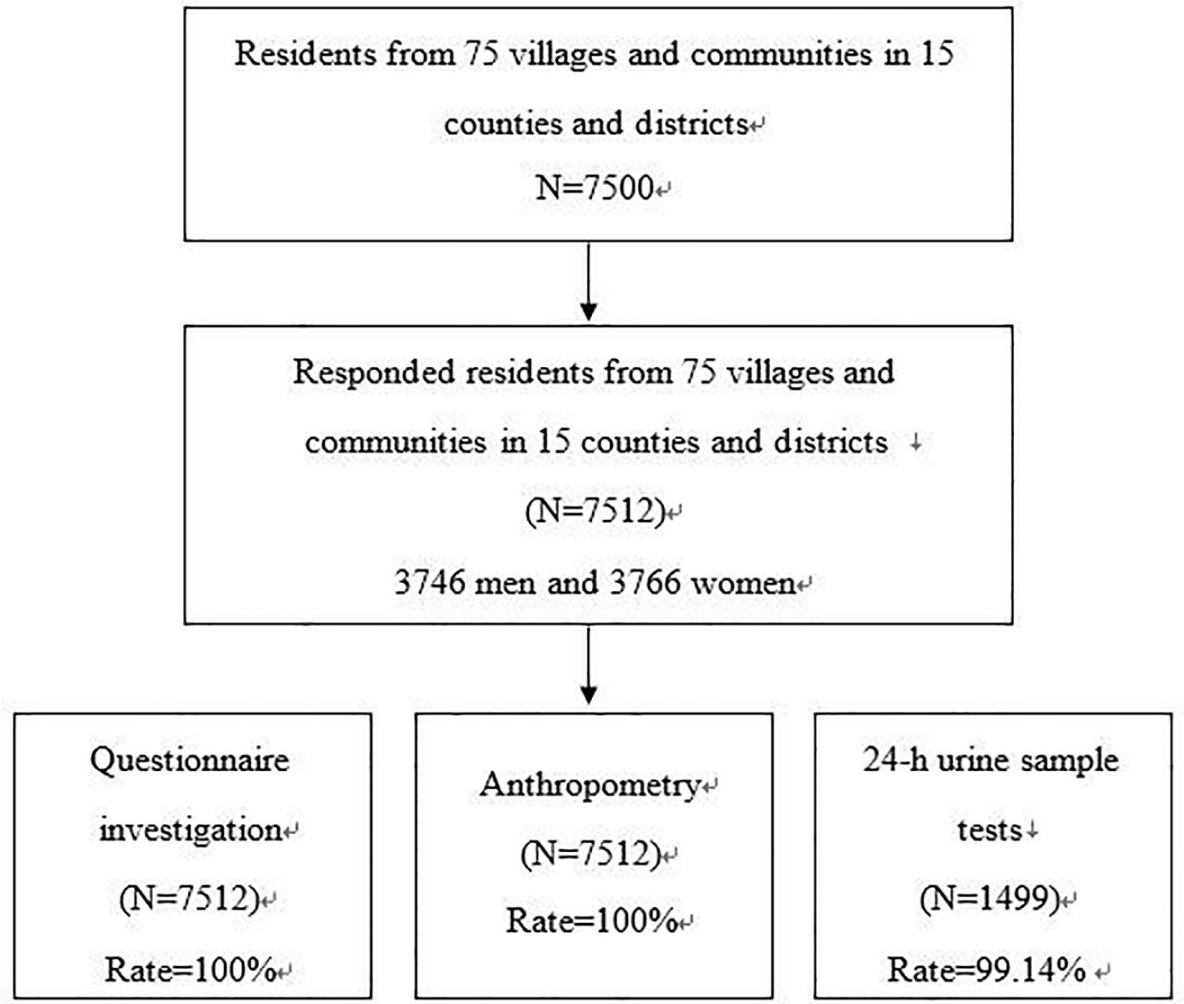
Study participants in the cross-sectional survey in Zhejiang Province of China in 2017.

The cross-sectional survey included questionnaire investigation, anthropometry, and biological sample tests for all respondents. One thousand five hundred and twelve participants were asked to collect 24-h urine, and laboratory tests determined the amounts of the chemicals in the urine. The details of sampling procedure and main measures are shown in Fig 1. Ethics approval was granted by Zhejiang Provincial CDC. Written participant consent was obtained from all participants via an active permission protocol based on the requirements of the Zhejiang Provincial CDC.

#### Questionnaire investigation

All the participants were invited to participate in a close-ended survey face-to-face by trained public health staff. The information on specific social demography, self-reported history of hypertension and diabetes, as well as the lifestyle habits of smoking, alcohol use, physical activity, and diet were collected. The survey also covered the respondents' knowledge of salt and hypertension, beliefs and attitudes about salt reduction, and information on salt reduction measures.

#### Anthropometry

Well-trained survey staff used the specified instrument or tool to measure physical measurements for the participants with standardized methods. The physical measurements included height, body weight, waist circumference, hip circumference, and blood pressure.

Height was measured with feet together without shoes and body upright. Bodyweight was measured by a calibrated electronic weight scale (HBF-371, Omron Corp., Japan) without heavy objects such as coats, shoes, and hats. Waist circumference was measured by tape measure on the plane of the midpoint of the line connecting the iliac crest to the lower edge of the rib. Hip circumference was measured by tape measure placed horizontally on the most prominent part of the gluteus maximus.

SBP and DBP measurements were performed according to internationally accepted measurement methods and quality control specifications with a calibrated electronic sphygmomanometer (HEM-7071, Omron Corp., Japan). Participants took the seat in a calm state, the upper arm was at the same level as the heart, and the lower edge of the cuff was 2.5 cm above the elbow. The measurement was carried out three times in succession at intervals of 1 minute, and the average value of the measurement results was taken. MBP (SBP + DBP divided by 2, mm Hg) was a measure of significant association with mortality from stroke mortality and ischemic heart disease mortality in comparison with SBP and DBP. [13]. Therefore, we also statistically analyzed this indicator.

#### Biological sample tests

At the end of the questionnaire investigation, the staff will issue a 3L standard urine collection container to 1,499 respondents and tell them how to retain 24-h urine. At the time of urine recovery, the staff recorded the beginning and end time of urine collection and assessed the completeness of 24-h urine collection by a standard questionnaire. For those who passed the assessment, the volume of urine was recorded using a measuring cup. 5 ml urine was pipetted into a cryotube and transported to a laboratory (KingMed Diagnostics Laboratory Inc., Hangzhou, China) using a refrigerator. The laboratory using standard test methods measured 24-h urinary sodium, potassium, creatinine, and microalbumin concentrations. The concentration was multiplied by the volume to obtain the content of each indicator.

Urinary sodium and potassium were measured with the ion-selecting electrode method by the testing equipment (C16000, Abbott Corp., America). Urinary creatinine was measured with the picric acid method (C501, Roche Cob as Corp., Switzerland). An incomplete urine collection was defined as either a 24-h urinary volume less than 500 ml or a 24-h urinary creatinine volume that was ±2 standard deviations (SD) outside of the sex-specific mean [14].

### Main variables definition

Body mass index (BMI, weight (kg) divided by height squared (m^2^)), is classified according to the following boundaries in the light of Chinese overweight and obesity guidelines[15]: BMI less than 18.5 is classified as lower weight, ranging from 18.5 to 23.99 as usual, ranging from 24 to 28 as overweight and BMI more than 28 is classified as obesity.

Current smoking is defined as continuous smoking for six months or more, with an average of 1 or more per day.

Hypertension was defined as mean SBP ≥ 140 mm Hg, mean DBP ≥ 90 mm Hg, or self-reported hypertensive patient or use of antihypertensive medication in the previous two weeks. Among respondents with hypertension, awareness of hypertension was defined as self-report a clear understanding that they have been diagnosed with hypertension by the medical institution, and treatment of hypertension was defined as self-report anti-hypertension medication usage. Finally, control of hypertension was defined as mean SBP < 140 mm Hg and mean DBP < 90 mm Hg [16].

### Quality control

The survey developed and implemented strict quality control measures to conduct quality control of the study and the collection of urine samples. The project team organizes relevant experts to demonstrate the investigation plan to ensure the scientific and rigorous design of the program, conduct unified training for the investigators, standardize the workflow, unify the measurement standards, and use uniform tools or laboratory testing methods. Quality control personnel supervise the investigation for verification and correction. The laboratory conducts quality control of daytime and intraday testing results.

### Data management and statistical analysis

Statistical analysis is performed using analytical methods of complex sampling. The sample weight was multiplied by the design weight and post-stratification weight. The design weight was multiplied by the weight of cluster design, strata, and individual. The weight of the post-stratification weight was determined by the general population distribution of Zhejiang Province in 2016 [17]. All measurement data were collected and summarized by panel computer issued uniformly. Statistical analyses were conducted using SAS software 9.4 (SAS Inc., Cary, North Carolina, USA), with alpha set at <0.05. The survey results were expressed as mean ± SD. The weighted mean and proportion were estimated, and 95%CI was calculated. Demographic and health characteristics were compared across different regions or genders using t-tests and analysis of variance (ANOVA) for continuous variables and the Rao-Scott χ2 test for categorical variables.

## Results

### Demographics of respondents

7512 individuals (3746 men, 3766 women) participated in our survey. 39.94% lived in urban areas, and 60.06% lived in rural areas. The weighted mean age was 40.66 years old (SD, 0.21). The respondents are basically Han Chinese (99.35%). In terms of education level, colleges or universities with more than 12 years of education account for 27.28%, and urban areas are better than rural areas. 1780 male participants were current smokers, and the males smoking rate (44.41%) is much higher than females (0.45%). Based on BMI, the prevalence of overweight and obesity was 33.57% and 11.58%, respectively. All details of respondents’ characteristics are shown in Table 1.

**Table 1:** Characteristics of study participants (N = 7512) in Zhejiang Province of China in 2017.

Characteristic	Total		Urban		Rural	
	N	% (95% CI) ^a^	N	% (95% CI) ^a^	N	% (95% CI) ^a^
Sex						
Male	3746	51.28(49.58–52.99)	1485	49.64(46.19–53.01)	2261	51.96(50.02–53.89)
Female	3766	48.72(47.01–50.42)	1515	50.36(46.90–53.81)	2251	48.04(46.11–49.98)
Ethnicity						
Han	7364	99.35(99.19–99.50)	2905	98.96(98.66–99.24)	4459	99.51(99.33–99.70)
Other ^b^	145	0.65(0.50–0.81)	94	1.05(0.76–1.34)	51	0.49(0.30–0.67)
Education, y						
<9	2281	21.54(20.30–22.78)	618	11.74(9.76–13.73)	1663	25.58(24.05–27.11)
9–12	3608	51.17(49.47–52.88)	1432	45.73(42.30–49.16)	2176	53.41(51.48–55.35)
>12	1620	27.28(25.65–28.92)	949	42.53(39.06–46.00)	671	21.01(19.27–22.74)
Smoking status, by sex						
Men						
Never	1656	46.33(43.92–48.75)	776	54.94(50.09–59.79)	880	42.95(40.17–45.72)
Former	308	9.25(7.88–10.63)	82	5.23(2.90–7.56)	226	10.83(9.16–12.51)
Current	1780	44.41(42.04–46.79)	626	39.83(35.07–44.59)	1154	46.22(43.49–48.95)
Women						
Never	3716	99.37(99.04–99.69)	1508	99.45(98.54–100.00)	2208	99.33(99.07–99.59)
Former	4	0.18(0.00–0.46)	1	0.46(0.00–1.36)	3	0.06(0.00–0.13)
Current	45	0.45(0.27–0.63)	6	0.09(0.00–0.22)	39	0.61(0.36–0.86)
BMI						
Low weight	284	4.82(4.02–5.62)	108	4.49(2.98–6.01)	176	4.95(4.01–5.89)
Normal	3768	50.03(48.32–51.73)	1477	50.31(46.86–53.77)	2291	49.91(47.97–51.85)
Overweight	2590	33.57(31.96–35.18)	1055	34.27(30.98–37.56)	1535	33.29(31.46–35.11)
Obese	870	11.58(10.48–12.68)	360	10.92(8.89–12.96)	510	11.85(10.55–13.15)

^a^ Percentages were weighted to represent the total population of Zhejiang adults aged 18 to 69 years poststratified by age and sex.

^b^ Other ethnicities were Hui, Miao, Uyghur, Yi, Tujia and She.

### Blood pressure and awareness, treatment and control of hypertension

Overall, the weighted mean of SBP and DBP was 127.78 mm Hg (95%CI, 127.21–128.36) and 79.40 mm Hg (95%CI, 79.04–79.76). The weighted mean of MBP was 103.59 mm Hg (95%CI, 103.15–104.04). The results of the study emphasized that the SBP, DBP and DBP (131.64, 81.86 and 106.75 mm Hg) of male participants were higher than female participants (123.73, 76.81 and 100.27 mm Hg), and rural areas (129.55, 79.65 and 104.60 mm Hg) were higher than urban areas(123.50, 78.79 and 101.14 mm Hg).

After weight adjustment of the gender and age structure of the population in Zhejiang Province, the survey results showed that the weighted prevalence of hypertension in Zhejiang Province was 30.41%(95%CI, 28.91–31.91), males (35.78%) was much higher than females (24.76%), and rural areas (31.65%) are higher than urban areas (27.40%). The results indicated that the prevalence of hypertension of females varied widely between regions, which did not exist in males.

Among all the respondents, 2653 patients with hypertension accounted for 30.41% (95%CI, 28.91–31.91). For those classified as having hypertension, 43.55% (95%CI, 40.77–46.34) of them were aware of their Illness, 32.05% (95%CI, 29.49–34.61) of them were taking antihypertension medications, and only 14.48% (95%CI, 12.54–16.42) of the hypertension patients could control blood pressure to normal levels. For the awareness, treatment, and control of hypertension, females were better than males, and urban areas were better than rural areas. The specific data is shown in Table 2.

**Table 2:** Means of SBP, DBP, MDP and prevalence, awareness, treatment, control of hypertension in Zhejiang Province of China in 2017^a^.

Measure	Total ^b^				Urban ^b^				Rural ^b^			
	N	Mean (95%CI) ^c^	χ^2^	*Pr*	N	Mean (95%CI) ^c^	χ^2^	*Pr*	N	Mean (95%CI) ^c^	χ^2^	*Pr*
Systolic blood pressure, mm Hg												
Men **	3746	131.64(130.9–132.37)	19.72 ^d^	<0.01	1485	128.37(127.08–129.66)	17.30 ^d^	<0.01	2261	132.92(132.05–133.80)	13.19^ ^^d^	<0.01
Women **	3766	123.73(122.89–124.58)			1515	118.70(117.24–120.15)			2251	125.90(124.91–126.90)		
Total **	7512	127.78(127.21–128.36)			3000	123.50(122.45–124.54)			4512	129.55(128.88–130.23)		
Diastolic blood pressure, mm Hg												
Men	3746	81.86(81.36–82.36)	21.18 ^d^	<0.01	1485	81.58(80.61–82.56)	15.62 ^d^	<0.01	2261	81.97(81.38–82.55)	15.34^ ^^d^	<0.01
Women **	3766	76.81(76.33–77.29)			1515	76.04(75.14–76.93)			2251	77.14(76.57–77.71)		
Total **	7512	79.40(79.04–79.76)			3000	78.79(78.09–79.49)			4512	79.65(79.23–80.07)		
Mid blood pressure, mm Hg												
Men **	3746	106.75(106.16–107.33)	21.54 ^d^	<0.01	1485	104.98(103.89–106.06)	17.60 ^d^	<0.01	2261	107.45(106.76–108.13)	14.87^ ^^d^	<0.01
Women **	3766	100.27(99.64–100.90)			1515	97.37(96.24–98.49)			2251	101.52(100.78–102.26)		
Total **	7512	103.59(103.15–104.04)			3000	101.14(100.31–101.98)			4512	104.60(104.08–105.12)		
Prevalence of hypertension												
Men	1479	35.78(33.53–38.03)	53.49	<0.01	571	35.52(30.96–40.08)	31.21	<0.01	908	35.88(33.31–38.45)	25.08	<0.01
Women **	1174	24.76(22.85–26.68)			432	19.40(15.95–22.84)			742	27.08(24.79–29.37)		
Total *	2653	30.41(28.91–31.91)			1003	27.40(24.48–30.32)			1650	31.65(29.92–33.39)		
Awareness of hypertension												
Men	678	40.06(36.43–43.70)	9.52	<0.01	297	45.58(37.87–53.28)	9.2	<0.01	381	37.92(33.86–41.98)	3.77	0.052
Women **	651	48.86(44.60–53.12)			280	64.48(55.26–73.70)			371	44.03(39.36–48.71)		
Total **	1329	43.55(40.77–46.34)			577	52.31(46.24–58.39)			752	40.43(37.36–43.5)		
Treatment of hypertension												
Men	449	27.52(24.33–30.70)	18.87	<0.01	210	32.03(25.29–38.77)	16.49	<0.01	239	25.75(22.16–29.34)	8.07	<0.01
Women **	471	38.92(34.78–43.07)			216	55.19(45.86–64.52)			255	33.89(29.51–38.28)		
Total **	920	32.05(29.49–34.61)			426	40.29(34.5–46.07)			494	29.11(26.32–31.89)		
Control of hypertension												
Men *	191	12.35(10.00–14.70)	7.19	<0.01	103	17.05(12.06–22.05)	12.78	<0.01	88	10.51(7.86–13.16)	1.02	0.31
Women **	224	17.73(14.43–21.02)			121	34.34(25.29–43.38)			103	12.59(9.51–15.67)		
Total **	415	14.48(12.54–16.42)			224	23.22(18.48–27.95)			191	11.37(9.36–13.38)		

^a^ Values are percentage (95% CI), unless otherwise indicated.

^b^ Rao-Scott χ^2^ test was used for comparisons of rates between different characteristics, unless otherwise indicated.

^c^ Means and percentages were weighted to represent the total population of Zhejiang adults aged 18 to 69 years poststratified by age and sex.

^d^ T-test was used for comparisons of means of SBP, DBP and MBP between different genders.

* Indicates *Pr*<0.05 for trend across differences between different regions based on t-test or chi-square test.

** Indicates *Pr*<0.01 for trend across differences between different regions based on t-test or chi-square test.

### Urine indicators such as urinary sodium, urinary potassium and urinary microalbumin

A total of 1499 of the respondents underwent 24-h urine collection and testing. The weighted mean of 24-h urinary sodium was 165.52 mmol (SD, 2.92), and salt intake converted from urinary sodium was 9.68g (SD, 0.17). The weighted mean of 24-h urinary potassium was 37.07 mmol (SD, 0.67), as while the weighted ratio of sodium to potassium was 4.96 (SD, 0.10). We also tested the urinary microalbumin, and its content at 24 hours was 16.34 mg (SD, 2.25). The results emphasized that males’ indicators were higher than females’ except for urinary potassium, especially for urinary microalbumin. Urinary sodium and potassium in urban areas were higher than in rural areas, but urinary microalbumin was higher in rural areas. Urinary sodium and potassium in urban areas are higher than in rural areas, and urine microalbumin content is reversed. With the increase of age, the content of urinary sodium, potassium, and microalbumin increased first and then decreased. Urinary sodium reached the maximum in the 30–39 age group, and urinary microalbumin reached the pick in the 50–59 age group. All details of respondents’ urinary index are shown in Table 3.

**Table 3:** 24 h urinary sodium, potassium and microalbumin excretion in different populations of Zhejiang Province of China in 2017 ^a^.

24 h Urinary measure	N	Sodium (mmol/24 h)		Potassium (mmol/24 h)		24 h Na/K		Converted Salt(g)		Microalbumin (mg/24 h)	
		Mean ± SD ^b^	*Pr*	Mean ± SD ^b^	*Pr*	Mean ± SD ^b^	*Pr*	Mean ± SD ^b^	Pr	Mean ± SD ^b^	*Pr*
Sex											
Male	733	171.41±4.22	<0.01	35.96±1.01	<0.05	5.39±0.15	<0.01	10.03±0.25	<0.01	21.81±4.39	<0.01
Female	766	159.69±4.05		38.18±0.88		4.54±0.13		9.34±0.24		10.92±0.97	
Residence											
Urban	598	187.54±6.29	<0.01	42.03±1.33	<0.01	4.95±0.19	0.88	10.97±0.37	<0.01	15.24±5.00	0.68
Rural	901	155.93±3.02		34.91±0.78		4.97±0.12		9.12±0.18		16.82±2.38	
Age, y ^c^											
18–29	261	162.29±6.53	<0.01	35.69±1.41	0.19	5.10±0.25	<0.05	9.49±0.38	<0.01	9.63±1.64	0.44
30–39	291	178.60±6.68		38.22±1.57		5.16±0.21		10.45±0.39		16.12±3.60	
40–49	271	173.00±6.85		38.77±1.52		5.01±0.25		10.12±0.40		18.06±6.66	
50–59	329	157.54±5.26		36.04±1.38		4.79±0.17		9.22±0.31		22.15±7.11	
60–69	347	145.45±4.49		35.88±1.40		4.52±0.13		8.51±0.26		16.88±2.42	
Total	1499	165.52±2.92		37.07±0.67		4.96±0.10		9.68±0.17		16.34±2.25	

^a^ T-test was used for comparisons of means between different characteristics, unless otherwise indicated.

^b^ Means were weighted to represent the total population of Zhejiang adults aged 18 to 69 years poststratified by age and sex.

^c^ ANOVA was used for comparisons of means between different age ranges.

## Discussion

Hypertension has been recognized as a significant risk factor for cardiovascular disease, which has become a nationwide epidemic in China [18, 19]. Some research emphasized that longitudinal change from normotensive or prehypertensive levels to 140/90 mm Hg or higher could increase the risk of total and cardiovascular mortality [20]. The prevalence of hypertension is rising fast, yet it remains inadequately controlled. We aimed to analyze the current prevalence, awareness, treatment, and control of hypertension in the Zhejiang Province of China. Our cross-sectional survey used the method of multistage stratified random cluster sampling to obtain a representative sample of adults aged from 18 to 69 years. We adjusted the weight of the sampled population and used the age and gender structure of the Zhejiang population to standardize the rates and some indicators to obtain provincial-level results, and also facilitated vertical comparison. We classify according to the primary attributes such as region, age, and gender, and statistically analyze the prevalence, awareness, treatment, and control of hypertension in different populations, as well as urine indicators. These methods are consistent with similar studies [21] and are also conducive to the targeted implementation of hypertension prevention and control measures.

A large-scale episode from Zhejiang Provincial Center for Cardiovascular Disease Control and Prevention showed that the prevalence of hypertension was 24.59% weighted in cities of urban areas and five counties of rural areas in Zhejiang Province in 2013 [22]. Compared with that the standardized rates of hypertension prevalence in residents aged from 18 to 69 years old in neighbor Jiangsu province in 2014 was 33.0% [21], and the weighted prevalence of hypertension was 23.4% in Shandong province in 2010 [23], our results highlighted that there is a high hypertension prevalence rate in Zhejiang Province, which is a public health problem needed timely control. Our findings highlighted differences in the prevalence of hypertension between gender and region. Males were higher than females, whether in urban or rural areas, which is related to the fact that males smoking, overweight or obesity is more senior than females, and we all know that smoking and excessive BMI are directly related to high blood pressure. Because that the smoking rate of women in urban areas (0.09%) is much lower than that of women in rural areas (0.61%), the prevalence of hypertension for females in urban areas was lower than that in rural areas, yet it was not significant among males.

It may remind us that smoking or obese males are more likely to have high blood pressure. The advantages of high economic level, low obesity, and an excellent healthy lifestyle may be the main reasons why the prevalence of hypertension in urban females is lower than that of rural females[24], which is also worth exploring further research. The results suggested that medical workers should focus on rural areas and males when implementing measures to prevent and control hypertension, especially those with smoking, obesity, and unhealthy lifestyles. Health education should be strengthened to control risk factors for hypertension. Tobacco reduction and bodyweight control should be performed simultaneously with lowering blood pressure.

The awareness rate of hypertension in our study is significantly different between males and females in urban areas, and this difference also exists among females in different regions. There is an essential relationship with the overall educational level, economic level, health conditions, and health awareness of people in different regions, while men do not pay much attention to their health and lack of active treatment.

The overall awareness rate (43.55%) is higher than that of neighboring Shandong Province (34.50%)[23] and Jiangsu Province(31.40%)[25], indicating that medical workers screen for hypertension and inform them of the condition timely. The treatment rate of hypertension (32.05%) is higher than the Shandong Province (27.50%). The control rate of hypertension (14.48%) is lower than that of Shandong Province (14.90%) or Jiangsu Province (23.70%). As we all know that the primary measures of hypertension control are drug intervention and non-drug intervention. That higher awareness and treatment rate with a lower control rate of hypertension indicated that non-drug intervention in Zhejiang Province needs to be strengthened, especially to control the use of tobacco, regular exercise, and body weight control. The findings highlighted the low awareness, treatment, and control rate of rural or male populations, again suggesting that these groups are vital to achieving prevention and control of hypertension.

Dietary sodium intake has been linked to blood pressure, and salt reduction has been documented to control hypertension, with relate researches increasing throughout the world [26–28]. How to accurately measure the sodium intake content has always been a problem. 24-h urine collection remains the preferred tool for assessing salt intake when compared with reported methods based on timed spot urine samples[29], although validation of urine collection completeness is difficult. Studies exploring alternative methods of 24-h urinary sodium detection have begun since the 1980s[30]. Despite this, we still used 24-h urinary sodium as a basis for the assessment of salt excretion, and we also did a urine integrity test, even if we did not use a more accurate method vs. para-aminobenzoic acid recovery (referent) [31]. In our study, the results appeared that the weight mean of salt consumption converted from 24-h urinary sodium was 9.68g, which far exceeds the recommended level (6 g) according to the dietary guidelines of Chinese residents (2016) [32] or the recommended level (5g) that World Health Organization (WHO) has recommended in 2003 [33]. The respondents whose salt consumption was below 6g accounted for only 17.53%.

There are two main aspects of controlling the effect of sodium on blood pressure. One is to control sodium intake, and the other is to increase potassium intake when allowed, such as no kidney disease or heart disease. The survey found that the ratio of sodium to potassium in men is higher than that in women. This poor diet combined with smoking, overweight, or obesity leads to a lower prevalence and control rate of hypertension in men. The sodium and potassium intakes in cities are higher than those in rural areas, but the ratio of sodium to potassium is at the same level. The high prevalence of hypertension and low control rate in rural areas also prove that controlling hypertension is a comprehensive project, which requires various a combination of healthy lifestyle interventions.

Because of many risk factors for hypertension, the way to prevent and control hypertension in the future must be diversified. It is worth mentioning that salt reduction must be an essential and indispensable method. In this study, we designed a survey of knowledge, attitudes, and behaviors for salt reduction. We found that 66.47% of the respondents knew to eat less salt could lower blood pressure, and 88.49% of them had a belief in reducing salt intake, and only 58.06% of the salt-reducing behavior occurred, indicating that knowledge was not effectively translated into a specific behavior. This suggests that we should focus on strengthening the instillation of salt reduction reasons and addressing salt reduction barriers, or introducing motivational interviews to increase compliance that helps explain the difference of urinary sodium between females and males, urban and rural areas. Besides, we should take a simple and easy way to monitor salt intake effectively, and recommend a low salt diet significantly decreased SBP and DBP, which should be commended for hypertensive patients [34].

It is the first time we have used 24-hour urine in Zhejiang Province to conduct a large-scale epidemiological survey of hypertension. We have established strict workflow and quality control, which will help to obtain more accurate data. However, we also have some limitations. Firstly, we have limited survey points, and the sample size can be further expanded to ensure better representation. Secondly, our 24-hour urine is collected only once, and the sodium content has a certain degree of volatility. Besides, because the survey site lasts for a long time (several months), the measurement of blood pressure is affected by temperature, environment, and instrumentation, which may lead to measurement errors in blood pressure.

## Conclusions

The prevalence of hypertension in Zhejiang Province has been gradually increasing, and awareness and treatment of hypertension appeared in differences in different regions and genders. The consumption of salt in Zhejiang Province is at a relatively high level. We should further control the risk factors of hypertensive patients and adopt appropriate non-pharmacological interventions such as health education, self-management, salt reduction, and low- sodium diets, especially in rural areas or male populations with more unhealthy lifestyles.

## Supporting information

S1 Table Minimal data set. (XLS)

S1 File Healthy condition questionnaire. (DOCX)
