# Supplementary material for: Prevalence, awareness, treatment and control of hypertension and sodium intake in Zhejiang Province, China: A cross-sectional survey in 2017
Source: PLoS One. 2019 Dec 23;14(12):e0226756. doi: 10.1371/journal.pone.0226756 (PMC6927602; doi:10.1371/journal.pone.0226756)
Supplement: S1 File — (DOCX) [file pone.0226756.s002.docx]

**S2 File. Healthy condition questionnaire**

Part 1. Behavior and Life style

1. Smoking

B1.1 Have you smoked over 100 cigarettes?

1=Yes, 2=No, 9=Uncertain.

B1.2 Are you still smoking?

1=Yes, 2=No.

B1.2.1 How many cigarettes do you smoke on average every day (weekly)? (Pick one of two)

___count / day ___count / week

B1.2.2 How long have you been smoking (years)? ___

B1.2.3 Someone who lives or works with you smokes?

1=Yes, 1=No.

B1.3 Do you often inhale smoke (ie passive smoking) exhaled by smokers for more than 15 minutes/day?

1= almost every day 2=an average of more than 3 days per week 3=averaging 1 to 3 days per week 4=less than one day per week on average 5=No 9=Not clear

1. Drinking

B2.1 Do you drink? (including liquor, beer, wine, etc.)

1=Yes, 2=No.

B2.2 How often have you been drinking in the past 12 months?

1=Every day 2=5-6 days/week 3=3-4 days/week 4=1-2 days/ week 5=1-3 days / month 6=less than 1 day/month

B2.3 Drinking condition:

Frequency*, Times**, Alcohol volume

B2.3.1 Chinese liquor (≥42 degrees) ____ ____ ____

B2.3.2 Chinese liquor (<42 degrees) ____ ____ ____

B2.3.3 bear ____ ____ ____

B2.3.4 wine ____ ____ ____

B2.3.5 yellow wine ____ ____ ____

B2.3.6 Rice wine ____ ____ ____

Note: Frequency *: 0=Never, 1=Day, 2=Week, 3=Month, 4=Year.

Times**: Which corresponds to Frequency.

3. Physical activity

B3.1 The strength and length of your daily work (or occupation) physical activity over the past 12 months?

1=light 2=Medium 3=Heavy

B3.2.1 In general, what is the main way you go to work / going to school / going out to work?

1=Walking 2=Bicycle 3=Car (self driving) 4=Motorcycle (electric vehicle) 5=Bus/bus 6=Basic stay at home

B3.2.2 In general, how long does it take for you to accumulate back and forth (minutes)?

___min

B3.3 In the past 12 months, have you exercised in addition to your work?

1=Yes, 2=No.

B3.3.1 What is the main type of physical exercise (up to 2)?

1=Jogging 2=Qigong 3=Tai Chi 4=Dancing 5=Walking 6=Swimming 7=Mountaineering 8=Speed running 9=Ball sports 10=other

B3.3.2 How many times have you exercised in the past year?

1=Exercise every day 2=3 times a week and above 3=1-2 times a week 4=1-3 times a month

B3.3.3 What is the average time of each exercise?

1=<10 minutes 2=10 minutes~ 3=20 minutes~ 4=30 minutes~ 5=more than 1 hour
